# Persistent Obstructive Sleep Apnea Post-adenotonsillectomy in Children

**DOI:** 10.7759/cureus.63899

**Published:** 2024-07-05

**Authors:** Ola Alhalabi, Amal R Al-Naimi, Faisal Abdulkader, Mutasim Abu-Hasan

**Affiliations:** 1 Pediatric Pulmonology, Sidra Medicine, Doha, QAT; 2 Ear, Nose, and Throat, Sidra Medicine, Doha, QAT

**Keywords:** prevalence, polysomnography, pediatrics, adenoidectomy and/or tonsillectomy, persistent obstructive sleep apnea

## Abstract

Background

Childhood obstructive sleep apnea (OSA) is a common disorder in children mostly due to adenotonsillar hypertrophy. Therefore, adenotonsillectomy is the mainstay of treatment. However, the outcome of adenotonsillectomy is limited in some patients who develop persistent OSA (POSA). We aim to evaluate the prevalence, risk factors, and treatments of POSA in the pediatric population in Qatar.

Methodology

This is a retrospective review of medical electronic records of patients aged 1-18 years, who underwent adenoidectomy and/or tonsillectomy at Sidra Medicine (Doha, Qatar) between June 2017 and September 2022. Demographic, clinical, and polysomnography (PSG) data were collected. POSA was defined as the persistence of at least one of the following OSA symptoms: snoring, gasping, mouth breathing or witnessed sleep apnea during post-surgery clinic visits, and/or post-surgical diagnosis of OSA by PSG. The prevalence of POSA was defined as the number of patients who had persistent symptoms divided by patients who were followed at outpatient clinics (ENT/pulmonology) post-surgery. Risk factors for POSA were evaluated using multivariate regression analysis.

Results

A total of 410 patients (259 males and 151 females) underwent adenotonsillectomy during the study period. The average age at surgery was 3.6 ± 2.5 years. The majority of patients (85.9%) had no history of underlying medical conditions. The rest of the patients (14.1%) were diagnosed with chromosomal abnormalities or neuromuscular disorders. All patients (100%) had a history of snoring before surgery, and 32.4% of patients had a history of witnessed sleep apnea. A total of 52 patients had persistent symptoms four months post-surgery. POSA prevalence was estimated at 15.4%. Univariate analysis showed young age at the time of surgery (p = 0.015), history of asthma (23%, 12/52) (p = 0.002), allergic rhinitis (13%, 7/52) (p = 0.001), gastroesophageal reflux disease ((11%, 6/52) (p < 0.001), and genetic syndromes (17%, 9/52) (p < 0.005) as significant risk factors for POSA. Multiple regression analysis showed that syndromic disorders and allergic rhinitis were significantly correlated with persistent OSA (p = 0.021 and p = 0.000, respectively).

Conclusions

POSA is prevalent in children post-tonsillectomy and adenoidectomy, especially in patients with genetic syndromes and those with symptoms of allergic rhinitis. Future studies are needed to better define the condition and provide evidence-based diagnostic and therapeutic approaches.

## Introduction

Obstructive sleep apnea (OSA) is a common respiratory problem affecting approximately 2-4% of children as per questionnaire-based diagnosis [1]. Polysomnography (PSG) remains the gold standard diagnostic test for OSA in children, and diagnosis is made based on the apnea-hypopnea index (AHI) of more than one event/hour [2]. Upper airway obstruction due to adenotonsillar hypertrophy is the leading cause of OSA in children, and adenotonsillectomy is the mainstay treatment [3]. However, children with OSA may continue to experience symptoms of OSA post-adenotonsillectomy, especially children with genetic syndromes or other comorbidities [3]. Unfortunately, there is no universally accepted definition for persistent OSA (POSA) in children. Ersu et al. recently defined POSA as unresolved OSA post-surgery based on PSG with an AHI of >1.5 events per hour [4]. Using PSG to diagnose POSA may lead to its underestimation as the majority of patients are unlikely to have PSG post-treatment. Other studies have used the persistence of symptoms of OSA as reported by patients using validated questionnaires [5]. Using patient-reported symptoms, on the other hand, may lead to overestimation of POSA.

Several risk factors have been identified for POSA in children, including male gender, obesity, history of isolated adenoidectomy or isolated tonsillectomy, surgery done early in life (less than two years of age), asthma, and allergic rhinitis [3,6]. Lingual tonsillar hypertrophy is the most commonly identified anatomical cause of POSA [7]. Differences in the frequency of risk factors can lead to differences in POSA prevalence among different population cohorts.

Close follow-up of patients with OSA post-treatment is crucial for early identification and diagnosis of POSA. PSG post-treatment is the most objective method to confirm the diagnosis. However, PSG is not widely available, time intensive, and can be costly. Therefore, most treating physicians use clinical judgment in diagnosing patients with POSA. Once the diagnosis of POSA is confirmed, further assessment may be needed to identify treatable risk factors of POSA. In specialized centers, evaluation of upper airways might be required to identify possible upper airway obstruction and localize the level of the obstruction, especially in patients with genetic disorders. Cine-magnetic resonance imaging (cine-MRI) and drug-induced sleep endoscopy (DISE) are increasingly used by specialized centers for detailed and dynamic examination of the upper airways [8-10]. Once the cause of upper airway obstruction is identified, patients require further interventions such as lingual tonsillectomy, laser supra-glottoplasty, endoscopic correction of choanal atresia, tracheostomy, or maxillofacial surgery [11-13]. If surgical correction of airway obstruction is not possible, then continuous positive airway pressure (CPAP)/bilevel positive airway pressure (BiPAP) therapy or bypassing obstruction using a tracheostomy tube may be needed in rare cases.

This study aimed to investigate the prevalence of POSA in the pediatric population of Qatar, identify its risk factors, and review its treatments. Such an endeavor will help us identify differences from other cohorts, develop population-based approaches for the diagnosis of POSA, and establish effective diagnosis and management plans.

## Materials and methods

This retrospective chart review collected and reviewed the electronic medical records of all patients who underwent adenoidectomy and/or tonsillectomy between June 2017 and September 2022 at Sidra Medicine. Our institute at Sidra Medicine represents the only pediatric tertiary hospital in Qatar.

The clinical, surgical, radiological, PSG, and postoperative data were retrieved from the electronic medical charts. Retrieved data included age at adenoidectomy and/or tonsillectomy; gender; prematurity; nutritional status at the time of surgery; underlying medical conditions such as genetic disorders, neuromuscular diseases, or craniofacial anomalies; respiratory and gastrointestinal conditions such as asthma, allergic rhinitis, and gastroesophageal reflux; the reason for intervention, the type of procedure, and the need and type of respiratory support before surgery.

Prematurity was defined as being born with a gestational age (GA) of less than 37 weeks. The nutritional status was assessed using a Z‐score of body mass index (BMI) for age and gender according to the World Health Organization (WHO) charts. Appropriate or normal nutritional status was defined as a Z-score between -2 and +2. Malnutrition was defined as a BMI Z-score of less than -2. Obesity or overweight was defined as a Z-score of more than +2.

Additionally, PSG data before and after surgery if done were collected. The following PSG data were reviewed: AHI, average nighttime oxygen saturation, and average nighttime end-tidal carbon dioxide (EtCO_2_). All PSG studies were scored according to the American Association of Sleep Medicine (AASM) Staging and Scoring Manual V2.5 2018. According to the AASM criteria, an AHI score of 1-4.9 events/hour is considered mild OSA, 5-9.9 events/hour is considered moderate OSA, and more than 10 events/hour is considered severe OSA.

Any records of postoperative residual symptoms such as snoring, mouth breathing, gasping, or witnessed sleep apnea were retrieved from post-surgery follow-up clinic visit records at ENT and pulmonology clinics.

POSA was defined as the persistence of at least one of the following symptoms: loud snoring, mouth breathing, gasping, or witnessed apnea events and/or post-operative PSG study showing AHI >1.5 events/hour four months post-adenoidectomy and/or tonsillectomy.

The prevalence of POSA was defined as the number of patients who had persistent symptoms divided by the total number of patients who continued to follow at the outpatient clinics (ENT/Pulmonology) post adenoidectomy and/or tonsillectomy.

All performed investigations for upper airway evaluation such as DISE or Cine-MRI were also obtained. Any surgical (i.e., tracheostomy, lingual tonsillectomy, supra-glottoplasty, maxillofacial surgery) or non-surgical management (i.e., non-invasive respiratory support including CPAP, or BiPAP) of POSA were also recorded.

Statistical analysis was performed using SPSS (SPSS Inc., Chicago, IL, USA) pocket program. Demographic and clinical characteristics of the patients were compared. Quantitative data were presented as median and range, while qualitative data were demonstrated as frequency and percentage (%). The categorical variables were compared with the t-test. P-values <0.05 were considered statistically significant for all analyses.

The study was reviewed and approved by the Institutional Review Board of Sidra Medicine (approval number: 1958856, dated: 10/8/2023).

## Results

A total of 410 patients (259 males and 151 females) underwent adenoidectomy and/or tonsillectomy at Sidra Medicine during the study period. The average age at surgery was 3.6 ± 2.5 years. Only 7.3% of the studied population were born premature. The average (range) BMI z-score was 1.68 (-10.7 to 4.54). The majority of the patients (85.85%) were healthy without any underlying medical conditions. The rest of the patients had chromosomal abnormalities or neuromuscular disorders. The most common preoperative symptom and indication for adenoidectomy and/or tonsillectomy was snoring (100%), and only 32.43% had a concurrent witnessed sleep apnea. Adenoidectomy was done in 239 patients, 162 patients underwent adenotonsillectomy, and nine patients underwent tonsillectomy only. The demographic and clinical data of patients are shown in Table 1.

**Table 1:** Characteristics of the Patients with OSA (N = 410). *: Patient may have more than one of these associations. MPS = mucopolysaccharidosis; OSA = obstructive sleep apnea; FTT = failure to thrive; OME = otitis media with effusion; CPAP = continuous positive airway pressure; BiPAP = bilevel positive airway pressure

Patient characteristics	
Gender	
Male, N (%)	259 (63.17%)
Female, N (%)	151 (36.83%)
Age at procedure (in years)	
Mean ± SD	3.6 ± 2.5
Median (range)	2.9 (0.6- 18)
Gestational age	
Term, N (%)	381 (92.9%)
Pre-term, N (%)	30 (7.3%)
Nutritional status	
Normal, N (%)	337 (82.19%)
Malnourished (FTT), N (%)	25 (6.09%)
Obesity/Overweight, N (%)	48 (11.7%)
Underlying condition	
Normal, N (%)	352 (85.85%)
Chromosomal disease, N (%)	20 (4.87%)
Achondroplasia, N	8
Trisomy 21, N	9
MPS, N	2
Crouzon, N	1
Pierre Robin, N	3
Neurological impairment, N (%)	12 (2.92%)
Neuromuscular diseases, N (%)	2 (0.48%)
Others, N (%)	24 (5.85%)
Respiratory/Gastrointestinal association*	
None, N (%)	355 (86.58%)
Asthma, N (%)	42 (10.24%)
Allergic rhinitis, N (%)	17 (4.15%)
Gastroesophageal reflux, N (%)	14 (3.41%)
Reason for intervention *	
Snoring, N (%)	410 (100%)
Mouth-breathing, N (%)	280 (68.29%)
Sleep disturbance, N (%)	196 (47.8%)
Apnea, N (%)	133 (32.43%)
Hearing/OME, N (%)	7 (1.7%)
Type of procedure	
Adenoidectomy, N (%)	239 (58.29%)
Tonsillectomy, N (%)	9 (2.19%)
Adenotonsillectomy, N (%)	162 (39.51%)
Respiratory support before surgery	
No, N (%)	402 (98.04%)
Yes, N (%)	8 (1.95%)
Type of respiratory support before surgery	
CPAP, N	3
BiPAP, N	5

Postoperatively, 337 (81%) patients presented for clinical assessment in the ENT/pulmonary clinics. The postoperative assessment was performed at an average (range) interval of four (0.83-17) months. A total of 52 patients had persistent symptoms four months post-adenoidectomy and/or tonsillectomy with a prevalence of POSA of 15.4%, as shown in Table 2.

**Table 2:** Post-adenoidectomy and/or tonsillectomy follow-up (N = 337). *: Patient may have more than one symptom. SD = standard deviation; OSA = obstructive sleep apnea; POSA = persistent obstructive sleep apnea

Outpatient follow-up, N (%)	337 (81.2%)
Average interval between surgery and follow-up (months) ±SD	3.89 ± 2.4
Symptoms post-procedure	
None, N (%) (resolved OSA)	285 (84.5%)
Residual symptoms (POSA)	52 (15.4%)
Snoring, N	52
Mouth-breathing	33
Apnea	14
Disturbed sleep	21
Performed postoperative polysomnography, N (%)	14 (4.15%)
Patients with POSA who required a second surgical intervention, N (%)	19 (36.5%)
Adenoidectomy, N	6
Tonsillectomy, N	2
Adenotonsillectomy, N	11

A total of 337 patients (81% of all study patients) had at least one postoperative follow-up clinic visit by either ENT or pulmonary teams. The clinic visit occurred four (0.83-17) months post-surgery. A total of 52 (15.4%) patients had reported persistent symptoms of OSA postoperatively. Only nine patients of the 52 patients (17.3%) had postoperative PSG: three (33.3%) of these patients were confirmed to have mild OSA (mean AHI: 1.8 ± 0.5 events/hour), and six (66.6%) patients had severe OSA (mean AHI: 39 ± 47 events/hour).

In the univariate regression analysis, the most significant risk factors for POSA were young age at surgery (3 ± 2.5 years) (p = 0.015), asthma (p = 0.002), allergic rhinitis (p = 0.001), gastroesophageal reflux disease (p = 0.001) and syndromic disorder (p = 0.006 × 10^-^⁶) (Table 3).

**Table 3:** Univariate regression analysis of POSA risk factors. POSA = persistent obstructive sleep apnea; OSA = obstructive sleep apnea; GERD = gastroesophageal reflux disease

Risk factor	POSA	Resolved OSA	P-value
Male gender, N (%)	35 (76%)	217 (76%)	0.63
Age at procedure (3 ± 2.5 years), N (%)	52 (100%)	285 (100%)	0.015
Prematurity, N (%)	4 (7.69%)	20 (7.04%)	0.86
Genetic abnormality, N (%)	9 (17%)	11 (3.80%)	0.000000006
Asthma, N (%)	12 (23%)	25 (8.77%)	0.002
Allergic rhinitis, N (%)	7 (13%)	9 (3.15%)	0.001
GERD, N (%)	6 (11%)	7 (2.45%)	0.001

In the multiple regression analysis, underlying syndromic diseases and allergic rhinitis were significantly correlated with POSA (p = 0.021 and p = 0.000, respectively) (Table 4).

**Table 4:** Multiple regression analysis for risk factors of POSA. aOR = adjusted odds ratio; CI = confidence interval; POSA = persistent obstructive sleep apnea

Variable	P-value	aOR	CI lower	CI Upper
Syndromic disorder	0.000	11.342	4.221	30.479
Asthma	0.053	0.412	0.168	1.010
Allergic rhinitis	0.021	0.253	0.079	0.815

Within the patient cohort, 35 patients had confirmed sleep-related disorders via PSG before surgery. Three patients had mild OSA, with a mean AHI of 3.3 ± 1.4 events/hour; eight patients had moderate OSA with an average AHI of 6.9 ± 1.1 events/hour; and 24 patients had severe OSA with an average AHI of 27.5 ± 22.3 events/hour. Oxygen saturation levels were determined to average 90.40 ± 16.5%, while the average EtCO_2_ concentration was 40 ± 8 mmHg. In instances where patients had POSA, a subset of nine patients was subjected to postoperative PSG. Within this subset, three patients were confirmed to have mild OSA with an average AHI of 1.8 ± 0.5 events/hour, while six individuals were diagnosed with severe OSA with an average AHI of 39 ± 47 events/hour. The recorded average oxygen saturation was 95 ± 3.4%.

Five of the patients with underlying genetic syndrome had upper airway evaluation by ENT specialty using DISE. Among the patients with POSA, 19 (36.5%) patients needed repeated surgical interventions: six patients underwent revision adenoidectomy, two patients underwent tonsillectomy, and 11 patients underwent adenotonsillectomy.

## Discussion

This retrospective review of patients with POSA who underwent adenoidectomy and/or tonsillectomy for OSA is the first study to evaluate the prevalence, risk factors, diagnostic, and treatment approach of these patients in Qatar. Notably, 84% of all patients with OSA had reported complete resolution of sleep-related respiratory symptoms post-surgery. In comparison, Riva et al. reported only 25% complete resolution of OSA based on PSG data post-adenotonsillectomy in a cohort of 210 patients [14]. Other studies reported cure rates for all children undergoing tonsillectomy varying from 51% to 83% [15]. The prevalence of POSA in our cohort was 15.4%. The large variation in prevalence rates of POSA is more likely related to differences in defining POSA. Moreover, the differences in the frequency of POSA risk factors among different cohorts can contribute to these differences. Our study showed high rates of resolution of OSA post-surgery based mainly on symptoms as only a few patients underwent PSG preoperatively and even less postoperatively. On the other hand, Bhattacharjee et el. studied 578 non-syndromic cohorts and reported that 27.2% of patients had complete resolution of OSA post-adenotonsillectomy based on PSG results [16].

In our study, patients with an underlying syndromic disorder and allergic rhinitis had a significant risk of developing POSA. These findings are in agreement with previous studies. For example, Cohen et el. evaluated 1,176 syndromic and non-syndromic pediatric patients pre and post-adenoidectomy and/or tonsillectomy. OSA symptoms were resolved in 67.9% of patients [5]. The syndromic condition was a significant risk factor for persistent symptoms post-adenoidectomy and/or tonsillectomy.

In other studies, OSA severity, history of prematurity, male gender, and obesity were associated with high risk for POSA [16,17]. In our cohort, gender, history of prematurity, and obesity were not significant risk factors for POSA (p = 0.86, p = 0.63, and p = 0.34, respectively).

We found that 36.5% of our patients with POSA underwent revision of adenoidectomy and/or tonsillectomy. Duval et al. reported that repeated adenoidectomy was required in only 1.5% of a large cohort of 10,948 pediatric patients [18]. The estimated likelihood of repeat surgery was estimated to be 2.5 times higher in children younger than five years. The theories regarding the high rate of tubal tonsillar hyperplasia, regrowth of residual adenoid tissue, and extraesophageal reflux in younger children can help explain this increased risk for repeated surgery.

A study by Wootten et al. demonstrated the efficacy of employing DISE-directed multilevel sleep surgery in improving both the AHI and symptomatology among 31 children persistently afflicted with OSA, half of whom had Down syndrome [19]. However, at our center, a minority of patients underwent endoscopic evaluation of upper airway anomalies post-adenoidectomy and/or tonsillectomy, a practice predominantly preserved for syndromic patients.

The retrospective design of the study and the absence of objective PDG-based criteria to define POSA and considering that 19% of our cohort had no postoperative follow-up clinic visit can affect our estimation of POSA prevalence. In the future, postoperative in-person or virtual clinic visits where screening for POSA is obtained through the utilization of validated sleep-disordered breathing questionnaires, home sleep apnea tests, or oxygen saturation trend studies can assist in systematically assessing the incidence of POSA or at least determine the necessity for postoperative PSG to confirm the diagnosis and assess the severity.

**Figure 1:**
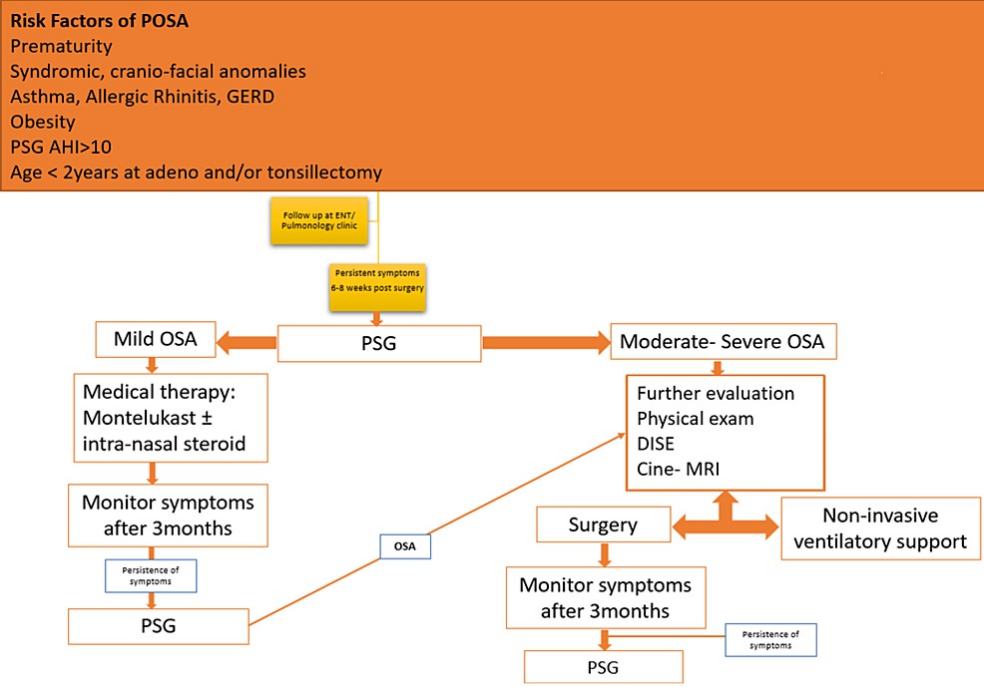
Diagnostic approach for the management of persistent obstructive sleep apnea. AHI = apnea-hypopnea index; Cine-MRI = Cine-magnetic resonance imaging; DISE = drug-induced sleep endoscopy; PSG = polysomnography; POSA = persistent obstructive sleep apnea

Once the diagnosis of POSA is confirmed, patients should be thoroughly evaluated for known risk factors of POSA. A high level of investigation for upper airways using DISE and/or Cine-MRI may be required in a subset of patients with POSA who are suspected to have upper airway obstruction to specify the level of obstruction and guide further surgical or medical therapy. Based on the results of our study and the accumulative data from the literature, we propose a diagnostic algorithm for the diagnosis and treatment of patients at risk of POSA [4,9,20], as shown in Figure 1.

## Conclusions

POSA post-adenoidectomy and/or tonsillectomy is a potentially serious health problem in the pediatric age group which is yet to be well defined. Variations in prevalence rates of POSA and its risk factors between different patient populations highlight this problem. Future efforts should be focused on providing an evidence-based definition of the disease and its risk factors as well as an effective treatment approach.
